# Associations of the magnesium depletion score and magnesium intake with diabetes among US adults: an analysis of the National Health and Nutrition Examination Survey 2011-2018

**DOI:** 10.4178/epih.e2024020

**Published:** 2024-01-10

**Authors:** Zhong Tian, Shifang Qu, Yana Chen, Jiaxin Fang, Xingxu Song, Kai He, Kexin Jiang, Xiaoyue Sun, Jianyang Shi, Yuchun Tao, Lina Jin

**Affiliations:** Department of Epidemiology and Biostatistics, School of Public Health, Jilin University, Changchun, China

**Keywords:** Diabetes, Magnesium depletion score, Magnesium

## Abstract

**OBJECTIVES:**

The magnesium depletion score (MDS) is considered more reliable than traditional approaches for predicting magnesium deficiency in humans. We explored the associations of MDS and dietary magnesium intake with diabetes.

**METHODS:**

We obtained data from 18,853 participants in the National Health and Nutrition Examination Survey 2011-2018. Using multivariate regression and stratified analysis, we investigated the relationships of both MDS and magnesium intake with diabetes. To compute prevalence ratios (PRs), we employed modified Poisson or log-binomial regression. We characterized the non-linear association between magnesium intake and diabetes using restricted cubic spline analysis.

**RESULTS:**

Participants with MDS ≥2 exhibited a PR of 1.26 (95% confidence interval [CI], 1.19 to 1.34) for diabetes. Per-standard deviation (SD) increase in dietary magnesium intake was associated with a lower prevalence of diabetes (PR, 0.91; 95% CI, 0.87 to 0.96). Subgroup analyses revealed a positive association between MDS ≥2 and diabetes across all levels of dietary magnesium intake, including the lowest (PR, 1.35; 95% CI, 1.18 to 1.55), middle (PR, 1.23; 95% CI, 1.12 to 1.35), and highest tertiles (PR, 1.25; 95% CI, 1.13 to 1.37; p_interaction_<0.001). Per-SD increase in magnesium intake was associated with lower diabetes prevalence in participants with MDS <2 (PR, 0.92; 95% CI, 0.87 to 0.98) and those with MDS ≥2 (PR, 0.91; 95% CI, 0.84 to 0.98; p_interaction_=0.030).

**CONCLUSIONS:**

MDS is associated with diabetes, particularly among individuals with low magnesium intake. Adequate dietary magnesium intake may reduce diabetes risk, especially in those with high MDS.

## GRAPHICAL ABSTRACT

[Fig f5-epih-46-e2024020]

## Key Message

The relationship between magnesium intake and risk is currently understudied in the field of diabetes prevention. The study found that magnesium deficiency is associated with diabetes risk, especially in people with low magnesium intake. Dietary magnesium supplementation may reduce risk and provide a new strategy for diabetes prevention. This study fills this knowledge gap and is important for scientific understanding of diabetes pathogenesis and epidemiological prevention and control.

## INTRODUCTION

Diabetes ranks among the most serious global health challenges of the 21st century [1]. Prior studies have generally indicated that diabetes arises from a complex interplay between genetic and environmental factors [2]. Nevertheless, mounting evidence points to dietary and lifestyle changes as the predominant drivers of the global diabetes pandemic [3,4]. Recent research has suggested that magnesium supplementation not only improves blood glucose levels in individuals with diabetes, but also enhances insulin sensitivity in populations at high risk for the disease [5].

Magnesium is an essential cofactor in numerous enzymatic reactions [6], playing a key role in maintaining glucose homeostasis and regulating insulin processes within the human body [7]. It is directly involved in insulin sensitivity and signaling in peripheral tissues and is vital for the activity of intracellular proteins that participate in insulin secretion in pancreatic beta cells [8]. Furthermore, insulin is an important regulator of magnesium ion (Mg^2+^) homeostasis, and insulin resistance can lead to decreased serum Mg^2+^ concentrations, thereby perpetuating a vicious cycle of type 2 diabetes and hypomagnesaemia [9]. Despite its importance, more than half of American adults do not consume adequate amounts of magnesium [10]. Persistent insufficient magnesium intake may lead to chronic or latent magnesium deficiency [11]. Such a deficiency can induce post-receptor insulin resistance and impaired cellular glucose utilization, further exacerbating insulin sensitivity impairment in individuals with diabetes [12]. Moreover, magnesium deficiency is widespread among patients with diabetes, with prevalence rates ranging from 13.5% to 47.7% [13], and lower serum magnesium levels appear to be associated with an increased risk of diabetes [14]. However, magnesium deficiency often does not present with specific clinical symptoms or signs, and standardized testing to accurately assess magnesium status is lacking [15].

Magnesium status in the human body depends on magnesium intake, absorption efficiency, and intestinal and renal excretion [16]. As previous studies have focused primarily on the impact of magnesium intake on diabetes, they have often overlooked magnesium status. The magnesium tolerance test is likely the most accurate method for evaluating magnesium status; however, its complexity has restricted its use in clinical settings [17,18]. Fan et al. [19] developed the concept of the magnesium depletion score (MDS) to predict magnesium deficiency by considering various factors that commonly influence the kidney’s capacity to reabsorb magnesium in the American population. Their findings indicated that the area-under-the-curve estimates for a model incorporating MDS alone, as well as for models of MDS adjusted for sex and age, were superior to those based on serum and urinary magnesium levels [19]. In comparison to other clinical indicators of magnesium deficiency, MDS has been shown to be more accurate and reliable.

To our knowledge, no prior research has examined the association between MDS and diabetes. Consequently, the objective of the present study was to explore this relationship, as well as the association between magnesium intake and diabetes. Furthermore, to prevent the excessive use of dietary supplements and to establish a foundation for the targeted prevention and treatment of diabetes, we sought to investigate the nature of this association in various subgroups.

## MATERIALS AND METHODS

### Data sources and study population

The National Health and Nutrition Examination Survey (NHANES) is a multistage, cross-sectional series of studies designed to evaluate the health and nutritional status of adults and children in the United States [20].

**Figure 1.**
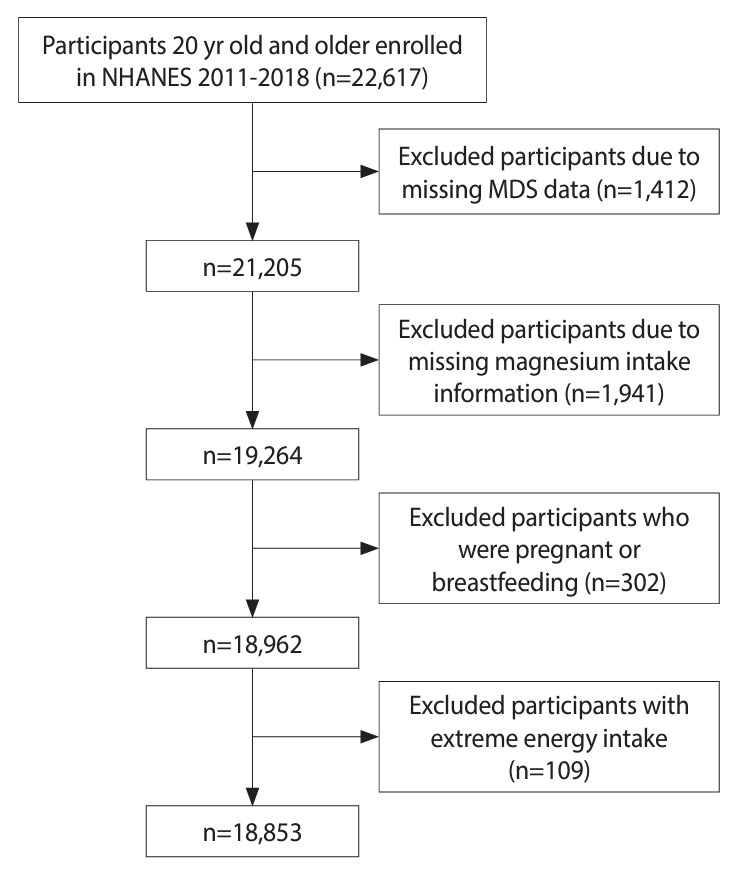
Flowchart illustrating the screening process used to select eligible National Health and Nutrition Examination Survey (NHANES) participants. MDS, magnesium depletion score.

The present study included 22,617 adult participants (aged 20 years or older) from the NHANES 2011-2018 dataset. We excluded individuals with missing data related to MDS (n = 1,412), those with no recorded dietary magnesium intake (n = 1,941), and pregnant or lactating females (n = 302). Additionally, we excluded females with a total energy intake below 500 kcal/day or above 5,000 kcal/day (n = 68), as well as males with an intake below 500 kcal/day or above 8,000 kcal/day (n = 41). After these exclusions, the study included a final sample of 18,853 participants (Figure 1).

### Exposure and outcome measures

MDS was constructed using 4 criteria: (1) current use of diuretics (assigned 1 point); (2) current use of proton pump inhibitors (1 point); (3) renal function as assessed using the Chronic Kidney Disease Epidemiology Collaboration equation [21,22], with 1 point allocated for an estimated glomerular filtration rate between 60 mL/(min· 1.73 m^2^) and less than 90 mL/(min· 1.73 m^2^), and 2 points for a rate below 60 mL/(min· 1.73 m^2^); and (4) heavy alcohol consumption, defined as more than 1 drink/day for females and more than 2 drink/day for males (1 point).

Subsequently, participants were categorized into 2 groups according to the calculated MDS: those with an MDS of less than 2 and those with an MDS of 2 or greater. Individuals presenting with an MDS of 2 or greater were deemed to be at elevated risk of magnesium deficiency [19].

Data regarding dietary magnesium intake were obtained from two 24-hour recall interviews conducted as part of the NHANES. The initial dietary recall interview took place at a mobile examination center, while the follow-up interview was conducted via telephone 3 days to 10 days later [23]. The dietary magnesium intake value used in this study was determined by averaging the 2 sets of 24-hour recall data. Energy-adjusted magnesium intake was then calculated using the residual method [24]. Regression analysis, subgroup analysis, and restricted cubic spline analysis were conducted using the energy-adjusted dietary magnesium intake values.

The U.S. Office of Dietary Supplements of the National Institutes of Health (http://ods.od.nih.gov/index.aspx), in conjunction with the Institute of Medicine, has established recommended daily allowances for magnesium intake that vary by sex and age. These recommendations are provided in Supplementary Material 1 to facilitate comparison with the magnesium intake levels reported in this study.

In our analysis, diabetes was defined by any of the following criteria: a self-reported diagnosis of diabetes, a fasting glucose level of 126 mg/dL or higher, an HbA1c value of 6.5% or greater, a glucose level of 200 mg/dL or above measured 2 hours after a 75-g oral glucose tolerance test, or any self-reported use of insulin or other diabetes medications.

### Covariates

This study incorporated various additional factors with the potential to influence the results, including age (categorized as under 50 or at least 50 years old), sex (male or female), race (non-Hispanic White or other), education level (up to high school/General Equivalency Diploma or beyond), marital status (married or other), poverty income ratio (PIR; 2.5 or lower, or higher than 2.5), and dietary intake levels of calcium, energy, fiber, and protein (averaged from two 24-hour recall interviews). Additionally, body mass index (BMI) (less than 30 or 30 kg/m^2^ and above), physical activity level (light or below, or moderate to vigorous), and smoking status (whether the individual had smoked at least 100 cigarettes in their lifetime [yes or no]) were considered.

### Statistical analysis

Participant characteristics were described using sampling weights that were based on the NHANES weight selection criteria. Categorical variables were compared between the groups with and without diabetes using chi-square tests, while continuous variables were examined using Student t-tests. To estimate prevalence ratios (PRs) and 95% confidence intervals (CIs) for the associations of both MDS and magnesium intake with diabetes, log-binomial regression was utilized [25]. When convergence issues arose, a modified Poisson regression approach was adopted to compute the PR [26]. The subgroup analyses involved stratification based on energy-adjusted magnesium intake (categorized into tertiles), MDS (less than 2, or 2 or greater), BMI (less than 30 or 30 kg/m^2^ and above), sex (male or female), age (under 50 or at least 50 years old), and smoking status (whether the individual had smoked at least 100 cigarettes in their lifetime [yes or no]). Additionally, p-values for interaction were calculated. To examine the non-linear association between magnesium intake and diabetes, a restricted cubic spline model was applied after adjusting for all covariates. A total of 4 knots were placed at the 5th, 35th, 65th, and 95th percentiles of the magnesium intake distribution [27]. All statistical analyses were conducted using R version 4.2.2 (R Foundation for Statistical Computing, Vienna, Austria) and SPSS version 24.0 (IBM Corp., Armonk, NY, USA).

### Ethics statement

National Center for Health Statistics Ethics Review Board approved the protocols for the NHANES. These protocols included the requirement to obtain informed consent from all participants.

## RESULTS

Table 1 presents the basic characteristics of all study participants. The overall weighted prevalence of diabetes was found to be 14.5%. When compared with the non-diabetic group, participants with diabetes consumed less dietary magnesium (284.16 ± 4.00 vs. 306.99 ± 2.44 mg/day; p < 0.001); however, the difference in energy-adjusted magnesium intake was not statistically significant. The distribution of MDS among the participants with diabetes differed significantly from that of the participants without diabetes (p < 0.001). Furthermore, significant differences were found between the diabetic and non-diabetic groups in several demographic and health-related factors, including age, sex, race, smoking status, marital status, level of physical activity, education level, PIR, BMI, and dietary intake of calcium, energy, and protein.

The relationship between MDS and diabetes, as well as the association between per-standard deviation (SD) increase in dietary magnesium intake and diabetes, was examined using log-binomial regression or modified Poisson regression modeling. Model 1 was adjusted for age, while model 2 was adjusted for sex, age, and race. Model 3 was adjusted for sex, age, race, smoking status, physical activity, BMI, education level, marital status, PIR, total energy intake, calcium intake, fiber intake, and protein intake. Table 2 presents the associations between MDS and diabetes. In all 3 models, MDS of 2 or higher was significantly associated with diabetes relative to MDS of less than 2 (p < 0.001 for all). In model 3, we observed that relative to a score of below 2, the PR for diabetes among those with an MDS of 2 or higher was 1.26 (95% CI, 1.19 to 1.34) (Table 2). Table 3 illustrates that in both model 1 and model 2, dietary magnesium intake (measured by per-SD increase) was significantly associated with the prevalence of diabetes (p < 0.05 for all). This association remained significant in model 3 (PR, 0.91; 95% CI, 0.87 to 0.96).

**Figure 2.**
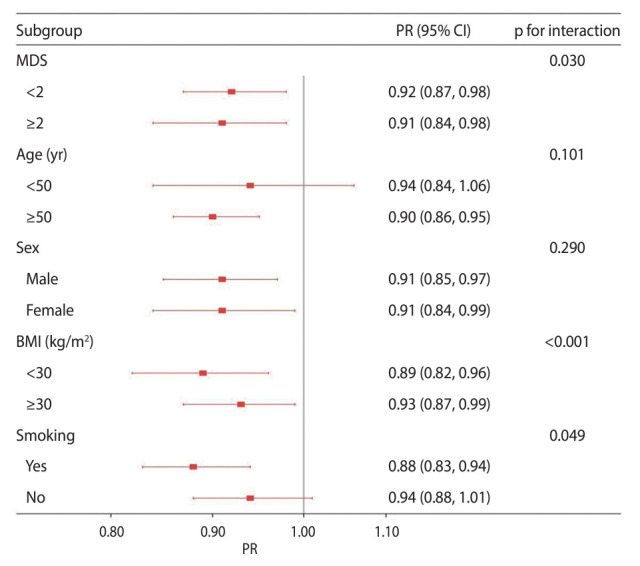
Subgroup analysis examining the associations between per-standard deviation increase in magnesium intake and diabetes. MDS, magnesium depletion score; PR, prevalence ratio; CI, confidence interval; BMI, body mass index.

**Figure 3.**
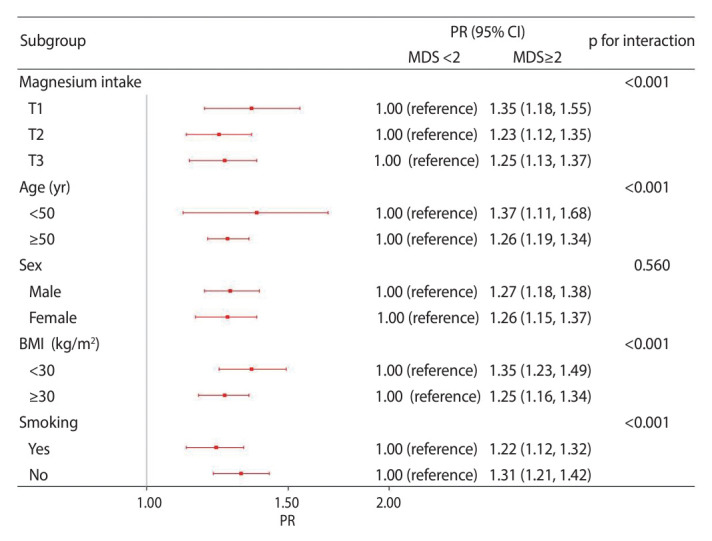
Subgroup analysis examining the associations between MDS and diabetes. The results of the subgroup analysis were adjusted for all covariates, except the effect modifier. MDS, magnesium depletion score; PR, prevalence ratio; CI, confidence interval; BMI, body mass index.

In the subgroup analysis, we adjusted for all covariates except the stratification factors. Our findings indicated a negative association between per-SD increase in dietary magnesium intake and the prevalence of diabetes in the group with MDS of 2 or higher (PR, 0.91; 95% CI, 0.84 to 0.98), as well as in those with MDS of less than 2 (PR, 0.92; 95% CI, 0.87 to 0.98; p_interaction_=0.030). We observed significant interactions between per-SD increase in dietary magnesium intake and both BMI (p_interaction_<0.001) and smoking status (p_interaction_=0.049) (Figure 2). In addition, MDS of at least 2 was positively correlated with diabetes prevalence across the spectrum of energy-adjusted magnesium intake, including the first (PR, 1.35; 95% CI, 1.18 to 1.55), second (PR, 1.23; 95% CI, 1.12 to 1.35), and third tertiles (PR, 1.25; 95% CI, 1.13 to 1.37, p_interaction_<0.001). Age, BMI, and smoking status showed significant interactions with MDS (p_interaction_<0.001 for all) (Figure 3).

In the stratified analyses, per-SD increase in magnesium intake was found to be negatively associated with diabetes, except among participants younger than 50 years and non-smokers. Furthermore, the analyses revealed that MDS was positively associated with diabetes across all subgroups.

**Figure 4.**
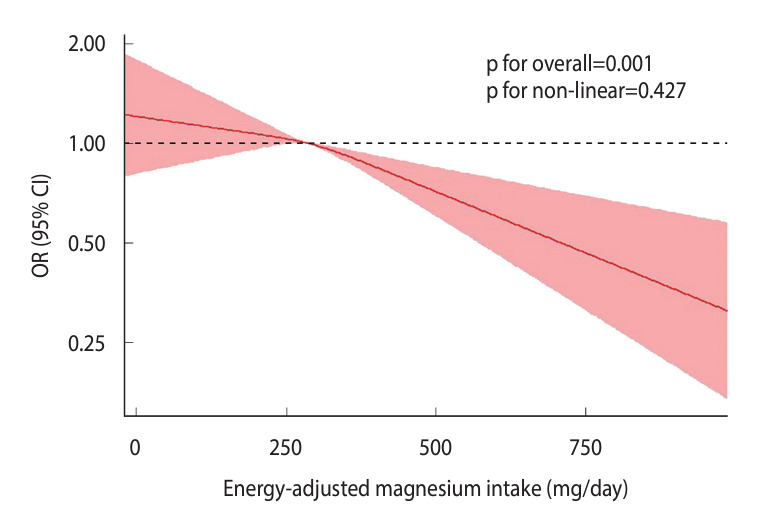
Examination of the non-linear association between dietary magnesium intake and diabetes, employing a random-effects model with the application of restricted cubic splines. OR, odds ratio; CI, confidence interval.

After adjusting for all covariates, we did not observe a significant non-linear dose-response relationship between magnesium intake and diabetes prevalence (Figure 4). The threshold for magnesium intake to confer a protective effect against diabetes was determined to be 288.01 mg/day.

## DISCUSSION

In this study, we noted a significant positive correlation between MDS and diabetes prevalence; however, this relationship was influenced by magnesium intake. Furthermore, we found that increased magnesium intake (measured per-SD) was negatively associated with diabetes, an association that was similarly impacted by MDS.

A systematic review previously demonstrated that magnesium intake exhibits an inverse dose-response association with the incidence of type 2 diabetes, suggesting that supplementation may be beneficial for glucose regulation in individuals with type 2 diabetes or those at high risk [28]. Despite this, more than half of American adults do not consume sufficient magnesium [10]. Persistent inadequate intake of this mineral can lead to chronic or latent magnesium deficiency [11]. Previous research indicates that magnesium deficiency is likely the most overlooked electrolyte imbalance in Western countries [29]. The most common method for assessing magnesium status is to measure the serum concentration [30]; however, only 0.3% of the body’s magnesium is detectable in serum [7]. As a result, individuals with chronic magnesium deficiency may not exhibit hypomagnesemia and can exhibit serum magnesium levels within the normal range [31]. In the absence of a commercially available and unequivocal biomarker for magnesium deficiency, it is important to pursue alternative methods for diagnosing this deficiency [15]. To date, no studies have thoroughly examined the effects of magnesium deficiency and magnesium intake on diabetes. Consequently, even in cross-sectional studies, it is essential to continue investigating the link between magnesium deficiency and diabetes, incorporating magnesium status to elucidate the role of magnesium intake in diabetes prevention.

The magnesium tolerance test is considered the most accurate method for evaluating magnesium status. However, its broad application is limited by its complex methodology and the potential for renal function to impact the results [18,32]. Consequently, we utilized MDS for the analysis in the present study. MDS considers 4 factors that influence the body’s magnesium stores: diuretic use, proton pump inhibitor use, renal function, and alcohol consumption. Moreover, MDS has demonstrated greater sensitivity and reliability than traditional methods in detecting actual magnesium deficiency in humans [19]. The findings of this study indicate that magnesium deficiency is linked to an elevated prevalence of diabetes, particularly when dietary magnesium intake is low. Additionally, the results suggest that the strength of the positive correlation between magnesium deficiency and diabetes may decrease as dietary magnesium intake rises. Furthermore, this correlation persists across populations of varying age, BMI, and smoking status.

Although considerable research has established a link between magnesium and diabetes, the molecular mechanisms by which a deficiency in magnesium leads to diabetes remain a topic of debate [33]. Studies have indicated that low magnesium levels can influence tyrosine kinase activity, disrupt post-receptor insulin signaling, and affect cellular glucose transport and utilization, culminating in insulin resistance and, eventually, diabetes [33]. Low magnesium levels have also been suggested to indirectly trigger the release of pro-inflammatory cytokines, thereby contributing to chronic inflammation and oxidative stress, which can lead to insulin resistance [8,34].

The Dietary Guidelines for Americans recommend a daily intake of 320 mg of magnesium for female and 420 mg for male [35]. However, over half of American adults consume less than the recommended amount of magnesium [10]. Oral magnesium supplementation has been shown to markedly reduce the prevalence of magnesium deficiency, decreasing it from 26.0% to 2.1% when serum magnesium levels below 0.70 mmol/L are used to define deficiency. However, to achieve steady-state serum magnesium concentrations, a minimum of 20 weeks of supplementation with at least 300 mg/day of magnesium is necessary [36]. The optimal daily dose of magnesium for diabetes prevention remains unclear. A meta-analysis by Xu et al. [37] suggested a non-linear relationship between magnesium intake and type 2 diabetes risk, proposing that a daily intake of 300 mg of magnesium may represent an effective level for combating type 2 diabetes. However, a separate meta-regression analysis of a cohort study revealed no evidence of a non-linear relationship between magnesium intake and diabetes risk [38]. We similarly did not observe a non-linear relationship between magnesium intake and diabetes (Figure 4). The discrepancy between our findings and those of Xu et al. [37] may be due to the use of energy-adjusted dietary magnesium intake in the present study.

When examining magnesium intake, it is also important to consider the body’s magnesium status and capacity for renal reabsorption. In a study of the bioavailability of magnesium supplements, Kappeler et al. [39] found that once the body’s storage capacity is saturated, any additional absorbed magnesium is not retained, but is rather excreted via the kidneys. As Figure 2 shows, MDS of less than 2 was associated with a smaller protective effect against diabetes with each SD increase in magnesium intake. This may be explained by the findings of Kappeler et al. [39]. In the present study, participants with MDS values of less than 2 were not considered to be at high risk for magnesium deficiency. Therefore, these individuals may not be deficient in magnesium, and increasing their magnesium intake may not result in additional storage within the body. To avoid the excessive use of supplements, we advise screening for magnesium deficiency in those at high risk before considering oral magnesium supplementation as a therapeutic option. As shown in Figure 3, the association between MDS and diabetes remained consistent across subgroup analyses for magnesium intake, age, sex, BMI, and smoking. However, the PR for the association between MDS and diabetes was comparatively low in the T2 group (PR, 1.23; 95% CI, 1.12 to 1.35) and the T3 group (PR, 1.25; 95% CI, 1.13 to 1.37; p_interaction_<0.001) regarding magnesium intake. This finding may be partially due to adequate magnesium intake influencing magnesium status. Additionally, the small number of diabetes cases in the T3 group may have impacted the results and conclusions. Therefore, we strongly recommend that future research focusing on the link between magnesium deficiency and diabetes emphasize the importance of dietary magnesium intake.

Despite the insights provided by the present study, it had certain limitations. First, its cross-sectional design precluded the establishment of a causal link between magnesium intake or MDS and diabetes. Furthermore, we relied on two 24-hour dietary recall interviews to estimate dietary intake, a method that is susceptible to recall bias.

In summary, a positive association was observed between MDS and diabetes prevalence, while magnesium intake displayed a negative association with diabetes. In both MDS subgroups (MDS ≥ 2 and < 2), per-SD increase in magnesium intake was associated with a reduced likelihood of diabetes. Furthermore, MDS maintained a positive association with diabetes across varying levels of magnesium intake.

## Figures and Tables

**Figure f5-epih-46-e2024020:**
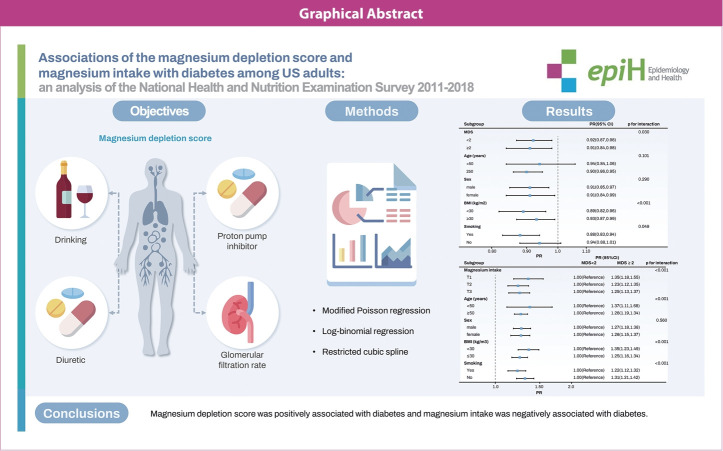


**Table 1.** Summary of participant characteristics by diabetes status

Characteristics	Non-diabetes	Diabetes	p-value^1^
Total	15,143 (85.5)	3,710 (14.5)	
Magnesium intake (mg/day)	306.99±2.44	284.16±4.00	<0.001
Energy-adjusted magnesium intake (mg/day)	303.93±2.11	302.16±2.37	0.459
Calcium (mg/day)	971.79±7.91	903.31±15.93	<0.001
Energy (kcal/day)	2,129.74±9.95	1,934.68±25.67	<0.001
Dietary fiber (g/day)	17.32±0.17	16.72±0.29	0.040
Protein (g/day)	83.00±0.48	78.53±1.10	<0.001
Sex			0.090
Male	7,403 (49.4)	1,941 (52.2)	
Female	7,740 (50.6)	1,769 (47.8)	
Age (yr)			<0.001
<50	8,550 (58.9)	731 (21.7)	
≥50	6,593 (41.1)	2,979 (78.2)	
Smoking			
Yes	6,403 (42.5)	1,804 (49.4)	<0.001
No	8,731 (57.5)	1,903 (50.6)	
Race			<0.001
Non-Hispanic White	6,043 (65.4)	1,229 (59.3)	
Other race	9,100 (34.6)	2,481 (40.7)	
Physical activity			<0.001
Light or below	8,558 (51.6)	2,438 (59.8)	
Moderate to vigorous	6,578 (48.4)	1,266 (40.2)	
Marital status			<0.001
Married	7,393 (52.2)	2,043 (58.5)	
Other	7,750 (47.8)	1,667 (41.5)	
Education level			<0.001
High school/GED or below	6,263 (34.8)	1,961 (45.6)	
More than high school	8,870 (65.2)	1,744 (54.4)	
Ratio of family income to poverty threshold			<0.001
<2.5	7,571 (43.0)	2,120 (50.0)	
≥2.5	6,230 (57.0)	1,246 (50.0)	
BMI (kg/m^2^)			<0.001
<30	9,727 (65.3)	1,532 (36.1)	
≥30	5,291 (34.7)	2,121 (63.9)	
MDS			<0.001
<2	12,109 (79.5)	2,283 (61.7)	
≥2	3,034 (20.5)	1,427 (38.3)	
Tertile (T) of energy-adjusted magnesium intake			0.058
T1	5,177 (31.7)	1,106 (29.1)	
T2	4,926 (33.6)	1,359 (36.9)	
T3	5,040 (34.7)	1,245 (34.0)	

Values are presented as mean±standard deviation or number (%), reflecting the complex sampling design employed by NHANES. GED, General Equivalency Diploma; NHANES, National Health and Nutrition Examination Survey; BMI, body mass index; MDS, magnesium depletion score.

1 Using the Student t-test or the Pearson chi-square test.

**Table 2.** Associations between magnesium depletion score (MDS) and diabetes

Model^1^	MDS		p-value
	<2	≥2	
Model 1	1.00 (reference)	1.35 (1.27, 1.42)	<0.001
Model 2	1.00 (reference)	1.42 (1.34, 1.50)	<0.001
Model 3	1.00 (reference)	1.26 (1.19, 1.34)	<0.001

Values are presented as prevalence ratio (95% confidence interval).

1 Model 1: Adjusted for age; Model 2: Adjusted for sex, age, and race; Model 3: Adjusted for sex, age, smoking status, race, physical activity, body mass index, education level, marital status, poverty income ratio, total energy intake, calcium intake, fiber intake, and protein intake.

**Table 3.** Associations between per-standard deviation increase in magnesium intake and diabetes

Model^1^	PR (95% CI)	p-value
Model 1	0.97 (0.94, 0.997)	0.029
Model 2	0.97 (0.94, 0.996)	0.025
Model 3	0.91 (0.87, 0.96)	<0.001

PR, prevalence ratio; CI, confidence interval.

1 Model 1: Adjusted for age; Model 2: Adjusted for sex, age, and race; Model 3: Adjusted for sex, age, smoking status, race, physical activity, body mass index, education level, marital status, poverty income ratio, total energy intake, calcium intake, fiber intake, and protein intake.

## Supplementary materials

Supplementary Material 1. US RDA for magnesium based on age and sex (US Office of Dietary Supplements of The National Institutes of Health)
