# Supplementary material for: Associations of the magnesium depletion score and magnesium intake with diabetes among US adults: an analysis of the National Health and Nutrition Examination Survey 2011-2018
Source: Epidemiol Health. 2024 Jan 10;46:e2024020. doi: 10.4178/epih.e2024020 (PMC11099598; doi:10.4178/epih.e2024020)
Supplement: Supplementary Material 1. — US RDA for magnesium based on age and sex (US Office of Dietary Supplements of The National Institutes of Health) [file epih-46-e2024020-Supplementary-1.doc]

| **Supplementary Material 1. US** **RDA for magnesium based on age and sex** | | | | |
| --- | --- | --- | --- | --- |
| **(US Office of Dietary Supplements of The National Institutes of Health)** | | | | |
| **Age (yr)** | | **Male** |  | **Female** |
| 14–18 |  | 410 mg |  | 360 mg |
|  |  |  |  |  |
| 19–30 |  | 400 mg |  | 310 mg |
|  |  |  |  |  |
| ≥31 |  | 420 mg |  | 320 mg |

RDA, recommended dietary allowance.
